# Complete Genome Sequence of the Novel *Psychrobacter* sp. Strain AJ006, Which Has the Potential for Biomineralization

**DOI:** 10.1128/MRA.00986-20

**Published:** 2020-10-08

**Authors:** Jun Ho Lee, Pyung Cheon Lee

**Affiliations:** a Department of Molecular Science and Technology, Ajou University, Woncheon-dong, Yeongtong-gu, Suwon, South Korea; b Department of Applied Chemistry and Biological Engineering, Ajou University, Woncheon-dong, Yeongtong-gu, Suwon, South Korea; Portland State University

## Abstract

A novel *Psychrobacter* sp. strain, AJ006, was isolated from Antarctic soil. Its complete genome sequence consists of a single circular chromosome (3,032,533 bp; G+C content, 44.0%) and a single linear plasmid (49,070 bp; G+C content, 41.7%). Chromosomal genes encoding carbonic anhydrase and urease, key enzymes in a biomineralization process, were predicted.

## ANNOUNCEMENT

Antarctic soils are suitable habitats for microorganisms with diverse activities and metabolic functions (1, 2). Recently, a novel psychrotolerant *Psychrobacter* sp. strain, AJ006, was isolated from Antarctic surface soil samples near King Sejong Station on King George Island (62°14′45.4″S, 58°46′36.2″W). *Psychrobacter* strains are of industrial interest because they have diverse catabolic activities with potential applications in bioremediation (3, 4) and biotechnology (5, 6).

The strain was obtained by three rounds of single-colony isolation on lysogeny broth (LB; BD Difco, USA) plates and aerobically cultured in LB at 25°C for 24 h. Genomic DNA was extracted (genomic DNA kit; Macrogen, South Korea) with RNase A treatment. Genomic DNA was sequenced in single-molecule real-time (SMRT) cells via RS II SMRT technology (Pacific Biosciences, CA) and the HiSeq 2000 platform (Illumina, USA) by DNA Link, Inc. (Seoul, South Korea). Sequencing libraries were prepared using a SMRTbell template prep kit 1.0 (for PacBio RS II) and TruSeq Nano DNA kit (for Illumina). All software was utilized with default parameters unless stated otherwise. After subread filtering of the PacBio raw data (read quality, ≥0.75), 161,634 long reads (average length, 2,264 bp; *N*_50_, 36,359 bp; total size, 365,893,261 bp; genome coverage, >120-fold) were generated and *de novo* assembled using the wtdbg2 v2.5 assembler (7) (settings, “-g 4m -p 13 -k 15 -AS 2 -s 0.1 -L 5000”). Seven resulting contigs were merged by running the “circlator merge” command in Circlator (8), and overlapping regions at both ends of the contigs were trimmed to create unique stretches at both ends using Circlator (setting, “b2r_length_cutoff=20000”). Two final assemblies were corrected in Quiver of SMRT Analysis v2.3.0 for three cycles (9) and polished using Pilon v1.22 (10) (setting, “--fix bases”) with trimmed paired-end Illumina reads (5,820,223 reads totaling 513,162,324 bp; genome coverage, >169-fold), obtained from 2 × 101-bp paired-end reads (8,790,922 reads totaling 887,883,122 bp) in Sickle v1.33 (https://github.com/najoshi/sickle). The assembly statistics were calculated via the stats.sh script from BBmap v38.68 (https://sourceforge.net/projects/bbmap/). Genome annotation and gene prediction were conducted using the Prokaryotic Genome Annotation Pipeline (11).

The genome sequence of *Psychrobacter* sp. strain AJ006 comprises a single 3,032,533-bp circular chromosome with 44.0% G+C content and a single 49,070-bp linear plasmid with 41.7% G+C content. Annotation revealed 2,539 coding DNA sequences and 54 RNA genes (3 copies of rRNAs and 45 tRNAs). 16S rRNA gene sequence analysis revealed that strain AJ006 has 97.98% sequence similarity to Psychrobacter pacificensis NIBH P2K6^T^. A triacylglycerol lipase gene was predicted and located near a lipase secretion chaperone gene (lipase helper protein) (12). Furthermore, chromosomal genes encoding carbonic anhydrase and urease were predicted. In a biomineralization process, the two enzymes are important for microbe-induced calcium carbonate precipitation (13).

### Data availability.

This shotgun sequencing project was deposited in DDBJ/EMBL/GenBank under the accession numbers CP060390 (for the chromosome) and CP060391 (for the plasmid). The SRA/DRA/ERA accession numbers are SRR11746895 (PacBio) and SRR11746894 (Illumina). The BioSample and BioProject numbers are SAMN14846924 and PRJNA630856, respectively.
